# Authors’ response:Vitamin D supplementation for improving sperm parameters in infertile men: A systematic review and meta-analysis of randomized clinical trials

**DOI:** 10.1080/2090598X.2023.2203605

**Published:** 2023-04-26

**Authors:** Clarissa Tania, Edwin Raja Pardamean Lumban Tobing, Christiano Tansol, Patricia Diana Prasetiyo, Caesar Khairul Wallad, Timotius Ivan Hariyanto

**Affiliations:** a Department of Urology, Faculty of Medicine, Pelita Harapan University, Karawaci, Tangerang, Indonesia; b Department of Pathology, Faculty of Medicine, Pelita Harapan University, Tangerang, Banten, Indonesia; c Department of Urology, Fatmawati General Hospital, Jakarta, Indonesia; d Faculty of Medicine, Pelita Harapan University, Karawaci, Tangerang, Indonesia

**Keywords:** vitamin D, nutrition, infertility, andrology, urology

Dear Editor,

We have read with the great interest a letter to editor regarding our article titled ‘Vitamin D supplementation for improving sperm parameters in infertile men: A systematic review and meta-analysis of randomized clinical trials’ which was published in the Arab Journal of Urology [1]. We thank the authors of the letter for paying much attention and raising some concerns of our article that may help us explain further the results from our study.

The first concern raised by the authors of the letter is related to the variation in vitamin D doses, age, and infertility diagnoses provided in the Table 2 of our article [1]. We don’t think that there is great variation in the age of the participants since all of the included RCTs have the mean age of 32–34 years [1]. Although the aspect of infertility in the participants of included RCTs are varied, those participants can still be grouped under one diagnosis which is men infertility, therefore considered as homogenous population [1–3]. We also think that the variation of vitamin D doses in the included RCTs is not considered as major flaws since the purpose of our meta-analysis is to give initial clue for vitamin D efficacy in treating men infertility [1]. We still encourage further randomized clinical trials (RCTs) to look for the most optimum vitamin D dose for management of infertility in men.

The second concern raised by the authors of the letter is about the high heterogeneity in some of the outcomes of interest in our article, namely total sperm count, progressive sperm motility and sperm morphology (I^2^>75%) [1]. We have mentioned the existence of this high heterogeneity as the limitations of our study. Heterogeneity itself is not something that is prohibited in meta-analysis, in fact it is something that cannot be avoided and should be expected [4]. It would be surprising if multiple studies conducted by different teams in different places with different methods all ended up estimating the same underlying parameter [4]. Moreover, there are no standardized international guidelines regarding acceptable degree of heterogeneity [4]. As long as a meta-analysis provides robust description of the predefined eligibility criteria and the presented data is also correct, then heterogeneity should not become a barrier in conducting a meta-analysis [4]. We also used a random-effect model on all outcomes of interest in our study to incorporate heterogeneity between studies.

In conclusion, considering the various limitations that exist in our study, we still advise the readers to be careful when interpreting the results of our study. We encourage well-designed RCTs with larger sample sizes to confirm the results of our study.

## Data Availability

Data analyzed in this study were a re-analysis of existing data, which are openly available at locations cited in the reference section.
